# Increase Human Metapneumovirus Mediated Morbidity following Pandemic Influenza Infection

**DOI:** 10.1371/journal.pone.0034750

**Published:** 2012-04-04

**Authors:** Liora Regev, Tal Meningher, Musa Hindiyeh, Ella Mendelson, Michal Mandelboim

**Affiliations:** 1 Central Virology Laboratory, Ministry of Health, Chaim Sheba Medical Center, Ramat-Gan, Israel; 2 Faculty of Life Sciences, Bar-Ilan University, Ramat Gan, Israel; 3 Department of Epidemiology and Preventive Medicine, School of Public Health, Sackler Faculty of Medicine, Tel-Aviv University, Tel-Aviv, Israel; University of Hong Kong, Hong Kong

## Abstract

Human metapneumovirus (hMPV) is a recently discovered respiratory pathogen, infecting mainly young children. The infected patients suffer from influenza like symptoms (ILS). In Israel the virus is mainly circulating in February to March. Here we report on an increased rate of hMPV infection in the winter season of 2009–10. The 2009–10 infection had several unique characteristics when compared to previous seasons; it started around January and a large number of infants were infected by the virus. Genetic analysis based on the viral L and F genes of hMPV showed that only subtypes A2 and B2 circulated in Israel. Additionally, we have identified a novel variant of hMPV within subgroup A2b, which subdivide it into A2b1 and A2b2. Finally, we showed that the hMPV infection was detected in the country soon after the infection with the pandemic influenza virus had declined, that infection with the pandemic influenza virus was dominant and that it interfered with the infection of other respiratory viruses. Thus, we suggest that the unusual increase in hMPV infection observed in 2009–10 was due to the appearance of the pandemic influenza virus in the winter season prior to 2009–10.

## Introduction

Human metapneumovirus (hMPV) is a member of the paramyxoviridae family which also includes respiratory syncytial virus (RSV), measles virus, and mumps virus [1], [2]. The infection symptoms caused by hMPV are similar to those caused by RSV and patients infected with hMPV demonstrates symptoms ranging from upper respiratory tract infection to bronchiolitis and pneumonia that is often accompanied by high fever, myalgia, and vomiting [2]–[7]. It has been suggested that hMPV is responsible for 5–10% of acute respiratory tract infections in neonates and children [8]–[10]. In general, hMPV infections are mainly observed in young children, in the elderly [11], [12] and in immunocompromised adults [13]–[16].

hMPV isolates are classified by phylogenetic analysis into two major genetic lineages termed subtypes A and B and are further subdivided into four subgroups (A1, A2, B1, and B2) [8], [10], [17]–[23]. Subtype A is the most dominant one [8], [17], [20], [23], [24]. A recent report demonstrated the existence of novel sub-lineages within the hMPV subgroup A2, named A2a and A2b [18], [23], [25]. Another recent study demonstrated the existence of various subtypes in the B2 group [26].

Here we report on an increased rate of hMPV infection in Israel in the year 2009–10, one year following the appearance of the pandemic influenza virus. We show that the 2009–10 hMPV infection had unique characteristics and we report on the identification of new hMPV subtype that we named A2b2.

## Materials and Methods

### Ethics

The institutional review board (IRB) of the Sheba Medical Center approved this research (Helsinki Number 9155-11-SMC). The work described in this manuscript is a retrospective study performed on patients samples that were analyzed for the presence of various viruses as part of the routine tests performed in the Sheba Medical Center and no extra samples were obtained for this research. The retrospective analysis performed was anonymous. Due to all of these reasons informed consent (either written or verbal) was not required.

### Patients and samples

Respiratory clinical samples (nasopharyngeal swabs or aspirates) were collected from 1635 patients hospitalized at Sheba Medical Center, Israel, due to respiratory illnesses, during the winter seasons between November 2007 and May 2010. Samples were retrospectively tested for the presence of hMPV, influenza viruses A and B, RSV and Adenovirus using a panel of real-time reverse transcription-PCR (rRT-PCR) and by real time PCR (for Adeno only) as previously described [27], [28]. In 2009–10 samples were also tested for the presence of pandemic influenza A(H1N1)pdm09 by real-time PCR using *Taq*Man chemistry [29].

### RNA extraction

Viral genomic RNA was extracted from patients' sample by using either High Pure viral RNA extraction kit (Roche Diagnostics GmbH, Mannheim, Germany) or by using the NucliSENS easyMAG (BioMerieux, France).

### DNA Sequencing

Two different sets of primers were used. The first set of primers was used to amplify part of the hMPV L (polymerase) gene and was used for diagnosis of patient clinical samples. Phylogenetic analysis based on 171-bp long sequence derived from the hMPV L gene was performed as described in Regev at el. [30]. The second set of primers was used for DNA sequencing and phylogenetic analysis amplified a 527-bp fragment of the hMPV F (Fusion) gene, as described in Carr at el. [26]. RT-PCR was performed using QIAGEN OneStep RT-PCR kit (QIAGEN GmbH, D-40724 Hilden, Germany). RT-PCR products were sequenced using ABI PRISM Dye Deoxy Terminator cycle sequencing kit (Applied Biosystems, Foster City, CA). Reaction mixtures were analyzed on Applied Biosystems model 373 DNA automatic sequencing systems.

### Phylogenetic analysis

The Sequencher® 5.0 program (Gencodes Corporation, Ann Arbor, MI) was used to compare the nucleotide sequences. Phylogenetic trees were prepared by nearest neighbor joint analysis using Clustal X with 1000 bootstraps and trees were visualized using TreeView or NJ plot software.

## Results

### Increased infection of hMPV during 2009–10

**Figure 1:**
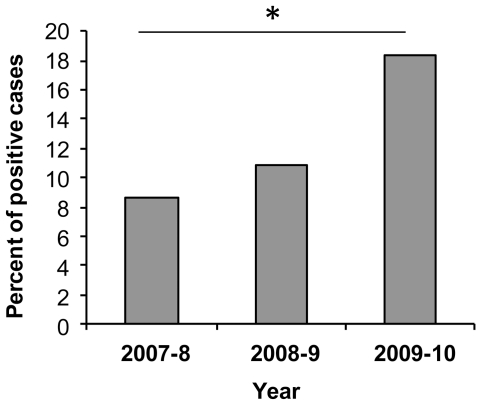
Percent of Hospitalized hMPV patients in Israel. Percent of patients positive for hMPV infections out of patients diagnosed for respiratory virus infections. The patients were hospitalized in the Sheba Medical Center, Israel, and three winter seasons 2007–10 are presented.

Our study was initiated because we observed a dramatic increase in the proportion of hMPV-infected patients that were hospitalized at the Sheba Medical Center during the 2009–10 winter season (between January to May). We have therefore decided to perform a retrospective examination on three winter seasons: 2007–8, 2008–9 and 2009–10. 1635 nasopharyngeal swabs or aspirates were collected and tested by qRT-PCR for hMPV infection. The hospitalized patients suffered from various symptoms; most of them had fever and cough. Some suffered from bronchitis and sore/red throat and small percentages of patients had also pneumonia (Table 1). As shown in figure 1, 15 of the 172 samples (8.7%) that were collected during the 2007–8 season, 58 of the 528 samples (11%) that were collected during the 2008–9 season and 172 of the 935 samples (18.4%) that were collected during the 2009–10 season were found to be hMPV positive (Figure 1). Significant (Chi square test) differences in the rates of hMPV positive cases were found between the winter seasons of 2009–10 and each of the two previous seasons, 2007–8 (P = 0.0027) and 2008–9 (P = 0.002), while the differences observed between 2007–8 and 2008–9 seasons, were not statistically significant (P = 0.48).

**Table 1:** Clinical characteristics.

Age (years)	Total (%)	Fever (%)	Cough (%)	Bronchitis (%)	Pneumonia (%)	Sore/Red throat (%)
**0–5**	47.3	50	47.8	50	66.6	34.7
**6–10**	10.7	11.4	10.8	0	0	14.3
**11–20**	5.4	5.7	5.4	0	0	8.2
**21–50**	19.3	17	18.5	7.1	8.3	28.6
**>50**	17.2	15.9	17.4	42.9	25	14.3
**Total (%)**	100	94.6	98.9	30	12.9	52.7

The table shows the ages of the various patients used in this study, the symptoms they suffer from and the percentages of patients in the various age groups that had these symptoms.

### Prevalence of hMPV infection in 2009–10

**Figure 2:**
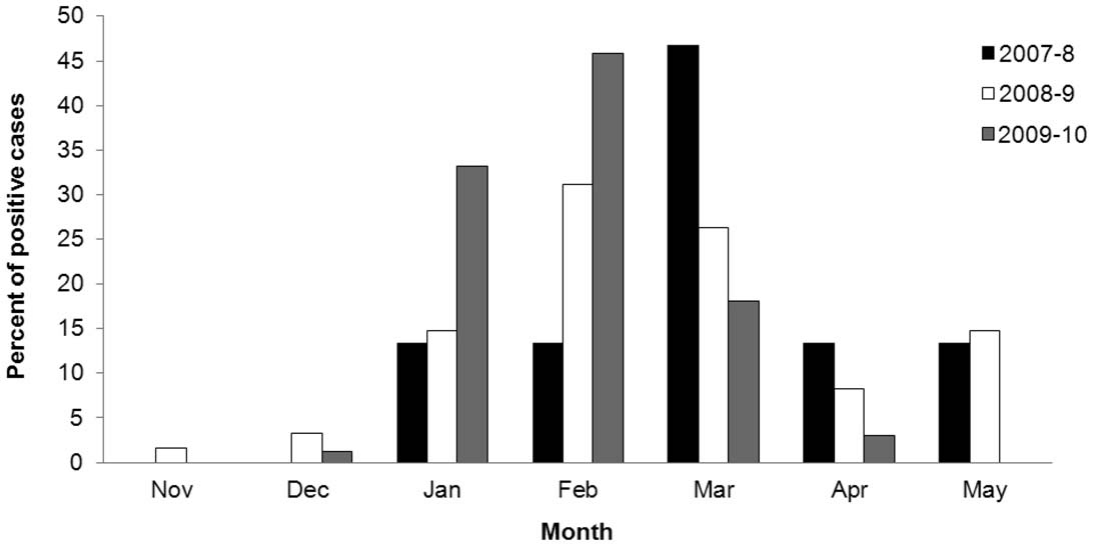
The hMPV infection in 2009–10 is observed early than usual. Precent of hMPV infected patients throughout the year. The figure shows themonthly distribution of hMPV positive cases diagnosed at the Central Virology Laboratory between November 2007 and May 2010.

To investigate the reasons for the increase in hMPV infections we next studied whether the 2009–10 infection manifest unusual characteristics. We initially examined the time in which infections with hMPV were detected. The analysis summarizes the percent of hMPV infected patients that were detected throughout the year. Normally, hMPV infections are frequent during the months of February and March. However, in 2009–10 the monthly distribution of hMPV infections was somewhat different from the two previous winter seasons. While in the 2007–8 and 2008–9 winter seasons a substantial number of cases were detected in February till March, in 2009–10 a significant number of hospitalized patients was already diagnosed in January and the percentages of hMPV infected patients were doubled (Figure 2).

### In 2009–10 infants were primarily infected with hMPV

**Figure 3:**
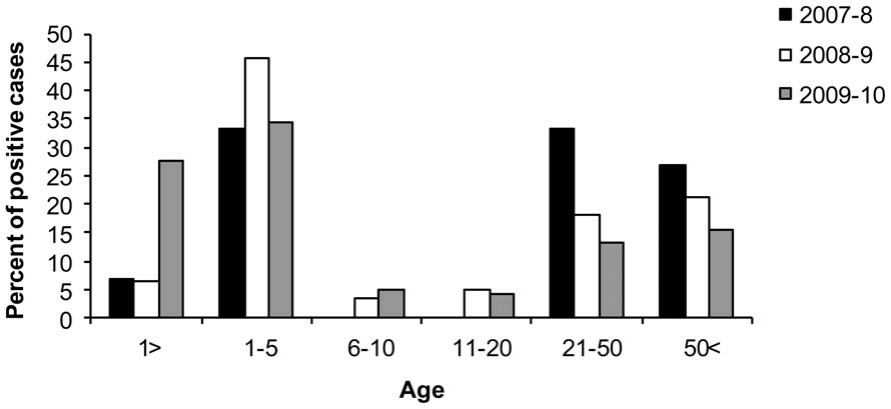
A large number of infants were infected with hMPV in 2009–10. The figure shows the percentages of the age distribution of the hMPV positive cases diagnosed at the Central Virology Laboratory between November 2007 and May 2010.

To further investigate the reasons accounting for the increase in hMPV infection observed in 2009–10 we investigated the age distribution of the patients infected in 2009–10 and compared it to the previous two winter seasons. As can be seen in figure 3, while in 2007–8 and in 2008–9 the ages of most of the hMPV-infected individuals range between 1–5 and from 21 and above, in the 2009–10 season, a large proportion of infants (age less than 1 year) were also infected (Figure 3).

It was previously reported that hMPV infections are associated with infections with additional viruses [31]–[33]. We therefore next investigated whether increased percentages of patients co-infected with several viruses will be observed in 2009–10. We found that while in 2007–8 and in 2008–9 13.3% and 12%, respectively, of the patients were co-infected with hMPV and additional viruses, in 2009–10 around 20% were co-infected with several viruses (data not shown). This difference however was not statistically significant. Thus, in 2009–10 hMPV infection was noticed in large percentages of patients, the infection was detected early than usual and infants were those who were primarily infected.

### Genetic analysis of the hMPV circulating strains

**Figure 4:**
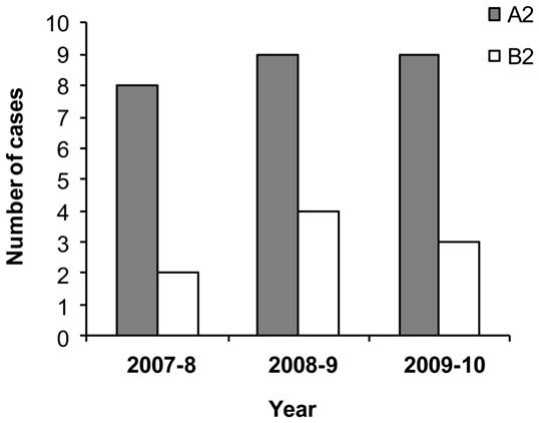
The A2 subtype is the dominant hMPV virus circulating in the country. The figure shows the number of cases (arbitrary selected) infected either with the A2 or the B2 subtypes of hMPV during the 3 winter seasons, from 2007–10. The various subtypes were determined by sequencing the L gene.

**Figure 5:**
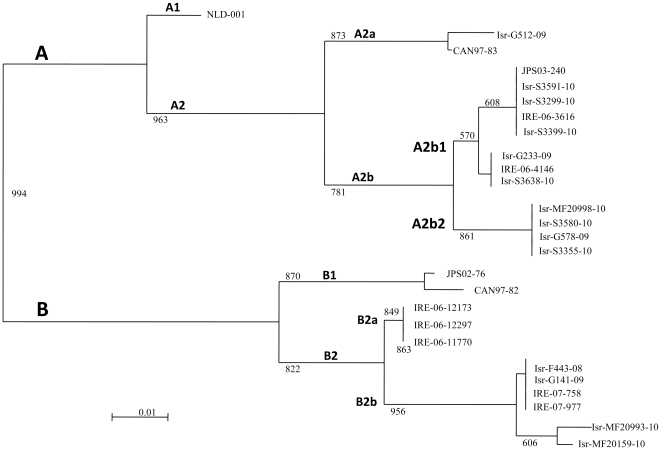
Identification of new hMPV subtype. The figure shoe a phylogenetic tree that was constructed based on the nucleotide sequences (111 bp) of the Fusion gene (F protein).

To test whether the unusual characteristics and the increase percentages of hMPV infection observed in 2009–10 was due to infection with a particular strain of hMPV that was present in the country during this year, we determine the hMPV types that circulated in Israel between 2007–10, by using RT-PCR. A portion of the L gene of hMPV [30] was sequenced in randomly selected clinical samples. As shown in figure 4, no major differences were observed in the circulating virus types, or subtypes in all 3 years of studied. Interestingly, the only two subtypes that were detected in Israel during these 3 seasons were A2 and B2, while the A1 and B1 subtypes were not detected. A phylogenetic analysis based on the F gene was used to determine whether new subtypes could be detected in Israel in the winter season of 2009–10. Fourteen arbitrary selected samples (obtained from both children and adults) that were obtained between the years 2007–10, were sequenced and as shown in figure 5, indeed only the A2 and the B2 subtypes were found, confirming our above observations (Figure 4). The B2b subtypes that was recently identified in Ireland [26], was also found in Israel (Figure 5). However, interestingly, new hMPV sub-lineages named A2b2 which splits the A2b subtype into two groups: A2b1 and A2b2, was identified by the phylogenetic analysis (Figure 5). This new sub-lineages however were already present in 2008–9 and therefore it is probably not the cause of the hMPV increase that occurred in 2009–10.

### Dominancy of pandemic influenza infection

Since no differences were observed between the various hMPV strains that circulated in the country in 2010 versus previous years we conclude that other factors are responsible for the increased hMPV infections observed in 2010.

**Figure 6:**
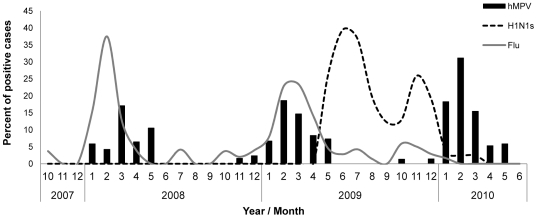
Respiratory viruses circulating in the country during the study period. The figure depicts percentages of patients hospitalized due to influenza-like symptoms that were found positive for infection with either influenza (A and B), with pandemic influenza (H1N1s) and hMPV between 2007–2010.

In April 2009 a new pathogen; the H1N1 pandemic influenza virus had circulated in the country and around the world. We therefore decided to test whether the appearance of the pandemic influenza virus in 2009 might have been responsible for the unusual characteristics of the hMPV infection observed in 2010. For that we have analyzed the incidence rates of laboratory detections of seasonal influenza, pandemic influenza and hMPV infections throughout the study period. As can be seen in figure 6, in 2007–8 and in 2008–9 the hMPV infections were observed almost concomitantly with seasonal influenza infections (Figure 6). A major change had occurred in April 2009 with the appearance of the pandemic influenza virus. The virus was present in the country until March 2010 (from January till March only few cases of pandemic influenza virus infections were detected) and two picks of infection were noted around June and around November 2009. Furthermore, the pandemic infection (H1N1) was dominant over other respiratory viruses, as infections with seasonal influenza (Figure 6) and other respiratory viruses (such as RSV, data not shown) could hardly be detected. Interestingly, the infection incidence of the pandemic influenza virus declined immediately prior to the appearance of the hMPV infections (Figure 6).

## Discussion

We report here on an increase hMPV infection that occurred in Israel in the winter season of 2009–10. In the years prior to 2009–10, the proportions of hMPV infections among hospitalized patients were similar to those reported by others [34]. In the winter of 2009–10, 18.4% of the patients were infected with hMPV. It should also be noted that we investigated here hospitalized patients only and thus the increased hMPV infection we observed might be only be the tip of the iceberg, given that most respiratory virus infections are mild (though not necessarily asymptomatic).

The hMPV infection in 2009–10 had several special properties; it appeared earlier than usual and a large proportion of infants less than 1 year old were infected. Infections with hMPV are frequently associated with a co-infection with another virus [31]–[33]. Indeed, around 20% of the hMPV infected patients in 2009–10 were co-infected with additional virus however this was not significantly different from previous years. We further discovered a new sub-lineage named A2b2. This observation, although interesting, cannot explain the hMPV morbidity observed in 2009–10, as this sub-lineage was already present in the population in the winter season of 2008–9 and most of the patients in 2009–10 were diagnosed to have the A2a1 subtype.

What could therefore be the reason for the increased hMPV infection? One possible explanation might be the appearance of the new pandemic influenza virus in 2009 [29] which circulated in Israel, having two infection peaks in June and in November 2009 [35]. Interestingly, we show that infection with the pandemic influenza virus was dominant over other respiratory viruses and that little or no other respiratory viruses, such as influenza and RSV, were active when the pandemic influenza virus was present (Figure 6 and data not shown). Strikingly, hMPV infections were noticed soon after the infection with the pandemic influenza virus had declined. Our assumption is that the pandemic influenza infection that emerged in the country in 2009 was dominant and thus it interfered with the infection of other respiratory viruses. Thus, once it declined, hMPV infections, which occur roughly at that period, were increased. We therefore suggest that it might be useful to vaccinate the population during infection of pandemic viruses not only against the circulating virus but also against additional respiratory viruses because, as we shown here, following pandemic influenza infections increased proportion of patients (especially young children) might be at danger of being infected with other respiratory viruses such as hMPV.
